# Supplementary material for: Effects of Alzheimer’s disease and formalin fixation on the different mineralised-iron forms in the human brain
Source: Sci Rep. 2020 Oct 5;10:16440. doi: 10.1038/s41598-020-73324-5 (PMC7536241; doi:10.1038/s41598-020-73324-5)
Supplement: Supplementary file 1 — Supplementary Information. [file 41598_2020_73324_MOESM1_ESM.pdf]

“Effects of Alzheimer’s disease and formalin fixation on the different mineralised-iron forms in the human brain”

Louise van der Weerd, Anton Lefering, Andrew Webb, Ramon Egli, and Lucia Bossoni

Supplementary material

Gender effect in the formalin-fixed samples

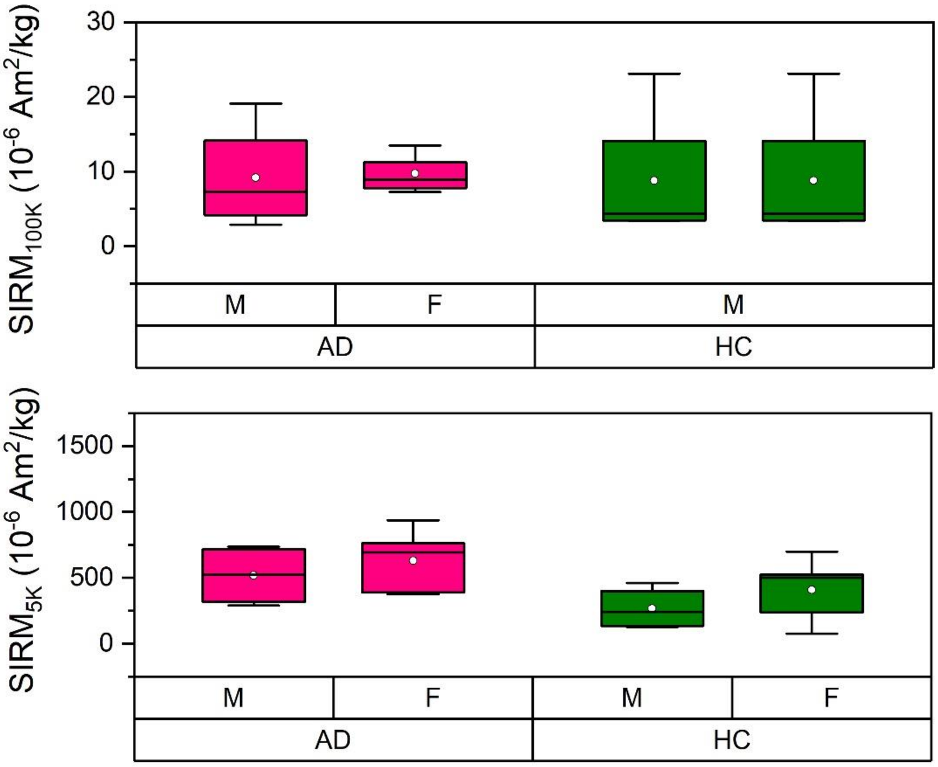

Figure 1S. Box-plot of the SIRM data at 100 K (top panel) and 5 K (bottom panel) stratified by gender and diagnosis. *Data were obtained from the formalin-fixed samples batch. The pink box-plots refer to the diseased cases (AD), while the green box-plots refer to the healthy controls (HC). ’M’ and ’F’ refer to the ’male’ and ’female’ group, respectively, as in the main manuscript.*

Validation of the use of the SIRM metric to quantify ferritin-iron in the brain

In our work, we used the saturated remanent magnetization (SIRM) parameter as a means to quantify molecular iron in the brain, under the assumption that a linear relationship holds between SIRM and the concentration of the molecular form of iron causing the IRM curve. Although previous authors [1] reported a linear relation between IRM values (measured at 5 K and 1 T) and total iron, a relation between SIRM and a specific molecular iron form has not previously been reported. Here below, we show that the SIRM metric obtained at 5 K is proportional to the amount of ferritin (-iron) in a brain sample with dispersed ferritin nanoparticles of known concentration.

A matrix-matched standard for brain ferritin was prepared by pooling brain material from subjects reported in this study, with lyophilized horse spleen ferritin purchased from Sigma-Aldrich (product number F4503, lot# SLBW3977), in increasing mass ratios of ferritin-to-brain (i.e. ferritin concentration).

IRM curves were acquired at 5 K, as described in this work, for each sample of the matrix. The results are shown in Fig 2S.

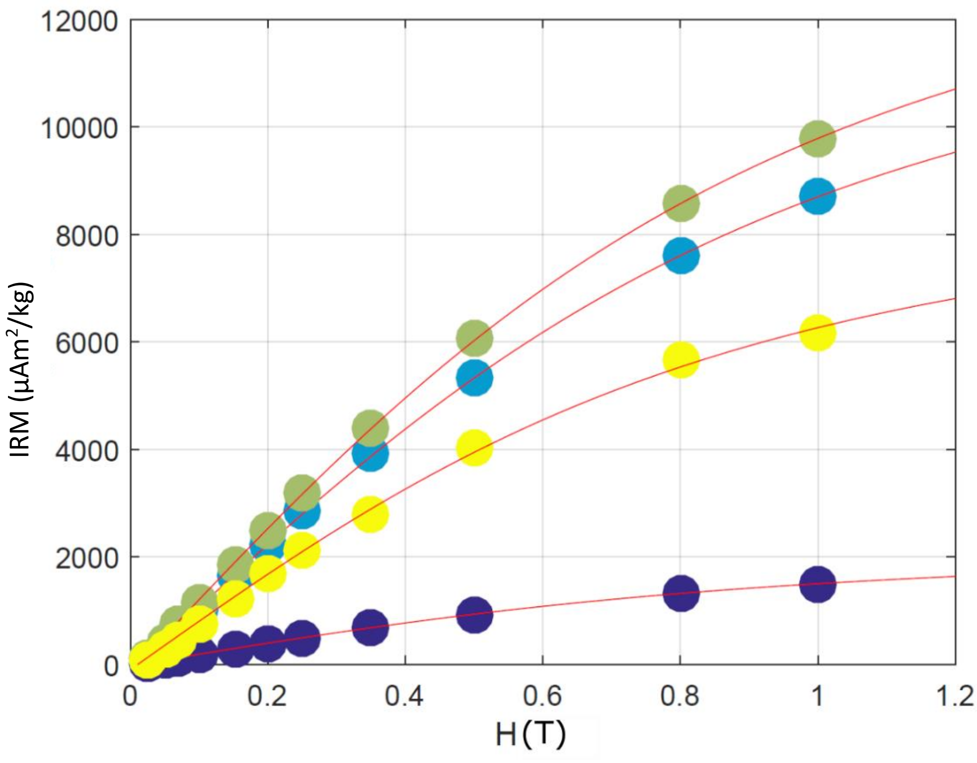

Figure 2S. IRM curves acquired on samples of the matrix-matched standard for brain ferritin. *Each color represents a different sample of the matrix. The red-solid line is the fit to the Langevin curve, as in the main text. The total mass of the samples was (dry weight): 0.141 g (blue circles), 0.144 g (yellow circles), 0.166 g (light blue circles) and 0.167 g (green circles).*

From the fit to the Langevin model, the SIRM parameter was obtained, and this was plotted against the ferritin concentration in the tissue (Figure 3S) and fitted to a first-order polynomial, with R^2=0.905.

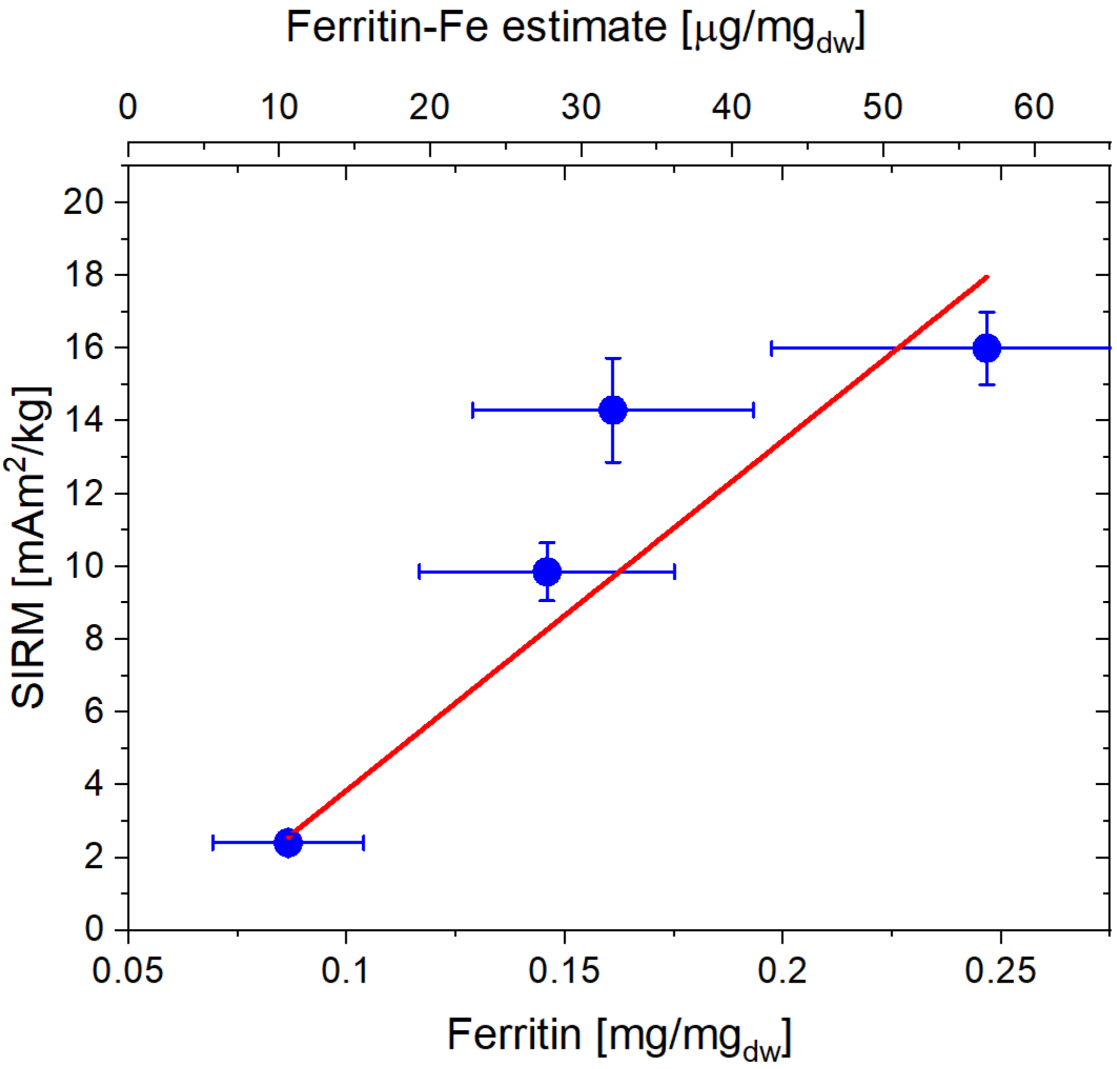

Figure 3S. SIRM value plotted against the ferritin concentration in the sample. *Upper x-axis is an estimate of the ferritin-iron content in the sample, assuming 2000 iron atoms per protein. The red line is the linear fit. Ferritin concentrations are referred to the dry weight of sample, i.e. ferritin powder and brain powder.*

Finally, we note that the large error bars on the ferritin concentration are caused by the pitfalls of sample preparation, which was exceptionally challenging due to the different nature of the lyophilized brain and ferritin powders, in terms on specific mass, charge and grain size.

[1] Naomi P. Visanji, Joanna F. Collingwood, Mary E. Finnegan, Anurag Tandon, Emily House and Lili-Naz Hazrati, Journal of Parkinson's Disease 3, 523–537 (2013) 523
